# A Multicenter Study of the Revogene C. difficile System for Detection of the Toxin B Gene from Unformed Stool Specimens

**DOI:** 10.1128/JCM.01510-19

**Published:** 2020-01-28

**Authors:** Michael J. Mashock, Matthew L. Faron, Karen C. Carroll, Christina Dang, Shawna Lewis, Hossein Salimnia, Paul Lephart, Vivian G. Loo, Bryan H. Schmitt, Stephen Young, Blake W. Buchan, Nathan A. Ledeboer

**Affiliations:** a Medical College of Wisconsin, Milwaukee, Wisconsin, USA; b Wisconsin Diagnostic Laboratories, Milwaukee, Wisconsin, USA; c Johns Hopkins Medical Institutions, Baltimore, Maryland, USA; d Detroit Medical Center University Laboratories, Detroit, Michigan, USA; e McGill University Health Centre, Montreal, Quebec, Canada; f Indiana University School of Medicine, Indianapolis, Indiana, USA; g Department of Pathology, University of New Mexico Health Sciences Center, Albuquerque, New Mexico, USA; h Tricore Reference Laboratories, Albuquerque, New Mexico, USA; Brigham and Women's Hospital

**Keywords:** *Clostridium difficile*, toxin B, clinical trials, diagnostics

## Abstract

Clostridioides difficile is the leading cause of diarrhea in hospitalized U.S. patients and results in over 400,000 cases of C. difficile infection per year. C. difficile infections have mortality rates of 6 to 30% and significantly increase health care costs, because of increased length of stay and increased frequency of readmissions due to recurrences. Efforts to reduce the spread of C. difficile in hospitals have led to the development of rapid sensitive diagnostic methods. A multicenter study was performed to establish the performance characteristics of the Revogene C. difficile test (Meridian Bioscience, Cincinnati, OH, USA) for use in detection of the toxin B (*tcdB*) gene from toxigenic C. difficile. The Revogene instrument is a new molecular platform that uses real-time PCR to detect nucleic acids in up to 8 specimens at a time. A total of 2,461 specimens from symptomatic patients that had been submitted for C. difficile testing were enrolled at 7 sites throughout the United States and Canada for evaluation of the assay. Each stool specimen was tested for the presence of the *tcdB* gene using the Revogene C. difficile test, and results were compared with those of the reference method, a combination of direct and enriched culture methods. Overall, the Revogene C. difficile test demonstrated a sensitivity of 85.0% (95% confidence interval, 80% to 88%) and a specificity of 97.2% (95% confidence interval, 96% to 98%). The Revogene C. difficile test, using clinical stool specimens for detection of *tcdB* in C. difficile, demonstrated acceptable sensitivity and specificity, with a short turnaround time.

## Abstract (precis)

Clostridioides difficile is the leading cause of diarrhea in hospitalized U.S. patients and results in over 400,000 cases of C. difficile infection per year. C. difficile infections have mortality rates of 6 to 30% and significantly increase health care costs, because of increased length of stay and increased frequency of readmissions due to recurrences. Efforts to reduce the spread of C. difficile in hospitals have led to the development of rapid sensitive diagnostic methods.

## INTRODUCTION

Clostridioides difficile is a sporulating, anaerobic, Gram-positive bacterium that is capable of colonizing the gut and causing infections under certain conditions. Patients exposed to antibiotics for long periods are at risk of C. difficile infection (CDI) as colonizing spores become vegetative due to lack of gut microbial flora and increases in bile salt concentrations (1). Replication of C. difficile can lead to increased release of the toxins TcdA and TcdB, causing severe diarrhea, loss of intestinal barrier function, colitis, and even death (1, 2). Currently, C. difficile is the most common nosocomial pathogen in the United States, with more than 400,000 cases of CDI and 29,000 deaths a year (3). CDI can quadruple the cost of hospitalizations (4) and increases U.S. health care costs by more than $1.5 billion per year (5). Active infections can be treated with the use of vancomycin, fidaxomicin, or even fecal transplantation, in a small number of cases; therefore, accurate diagnostics for active infections are necessary to improve patient care. However, because 2 to 17% of healthy patients can be colonized (2, 6), the use of clinical assessment criteria to guide proper testing or testing algorithms to differentiate between colonization and active infection is advised.

To optimize patient management, the European Society of Clinical Microbiology and Infectious Diseases recommends first performing a sensitive diagnostic procedure such as a glutamate dehydrogenase (GDH) assay or a *tcdB* nucleic acid amplification test, followed by toxin detection with either a toxin A/B enzyme immunoassay or a toxigenic culture (TC) (7). Molecular assays have high sensitivity and specificity, but the detection of a toxigenic C. difficile strain does not automatically indicate that patient treatment is required, because this can lead to unnecessary treatment of colonized patients (8, 9). Consequently, it remains crucial to clinically assess patients and to submit specimens only from patients at high risk of having CDI, to help avoid unnecessary testing. If proper clinical assessment is performed, based on preestablished institutional criteria, then the use of standalone molecular methods is sufficient (10). In addition, Infectious Diseases Society of America (IDSA) guidelines suggest that C. difficile testing should be performed only for patients presenting with recent unformed stools occurring ≥3 times in a <24-h period.

Here, we present a multisite investigational evaluation of the Revogene C. difficile test for use in detection of the toxin B gene from toxigenic strains of C. difficile. The Meridian Bioscience Revogene instrument is a real-time PCR (rtPCR)-based assay system that allows easy molecular detection of C. difficile in up to 8 specimens after a single heating step. In this study, a total of 2,461 residual stool specimens from 7 sites within the United States and Canada were enrolled for molecular detection of both C. difficile and *tcdB*.

## MATERIALS AND METHODS

### Collection of C. difficile specimens.

Residual, raw, unformed stool samples submitted to the clinical laboratory for C. difficile testing were enrolled at 7 geographically distributed clinical centers within Canada and the United States, i.e., Medical College of Wisconsin (Milwaukee, WI, USA), Sinai Health System (Toronto, ON, Canada), Research Institute of the McGill University Health Centre (Montreal, QC, Canada), Tricore Reference Laboratories (Albuquerque, NM, USA), Wayne State University School of Medicine (Detroit, MI, USA), Johns Hopkins University School of Medicine (Baltimore, MD, USA), and Indiana University School of Medicine (Indianapolis, IN, USA). All specimens were deidentified before enrollment and after completion of standard-of-care (SOC) testing. In total, 797 specimens were prospectively enrolled; 1,664 specimens were retrospective specimens that had been stored at –80°C. Microbiology Specialists Inc. (Houston, TX, USA) performed the reference testing. All sites obtained appropriate institutional review board approval or waivers, as specified by their local human subjects research committees.

### Toxigenic culture.

TC was performed at a single reference site (Microbiology Specialists Inc.). Specimens were shipped daily in anaerobic transport medium (Anaerobe Systems, Morgan Hill, CA, USA) and both tested directly and enriched within 4 days after collection. Direct TC testing involved inoculating the sample onto a standard cycloserine-cefoxitin-fructose agar (CCFA) plate (Anaerobe Systems), which was incubated for 48 h at 35°C to 37°C under anaerobic conditions. The CCFA plate was then examined for any colonies resembling C. difficile. Enrichment was performed like direct TC but included an additional incubation for 48 h at 35°C to 37°C in cycloserine-cefoxitin-mannitol broth with taurocholate and lysozyme, under anaerobic conditions. If direct culture identified C. difficile colonies, then enrichment testing was discontinued. Possible C. difficile isolates were grown in chopped meat carbohydrate (CMC) broth (Anaerobe Systems) and then identified using gas-liquid chromatography (GLC).

### Cell cytotoxicity neutralization assay.

All GLC-positive C. difficile isolates were tested for toxigenicity, from the same CMC broth, using a cell cytotoxicity neutralization assay, according to a Microbiology Specialists Inc. protocol (protocol no. ST040). In summary, cell-free culture filtrate was generated by centrifugation of CMC broth at 3,000 rpm for 1 h, filtered, and diluted 1:5 with sterile diluent. The diluted culture filtrate was further diluted with sterile diluent or C. difficile antitoxin (1:10 dilution) and incubated at room temperature for 30 min. Diluted specimen filtrate (with or without antitoxin) was then used to inoculate HFF-1 culture tubes (final dilution, 1:40), which were incubated for 48 h at 35°C in a rolling tube incubator. If cells in specimen filtrate with antitoxin had normal morphology but cells in specimen filtrate appeared rounded, then C. difficile toxin B was reported to be present.

### Revogene C. difficile testing.

Testing with the Revogene C. difficile assay was performed by following the package insert. Briefly, the stool specimens were eluted with a 5-μl loop into a buffer, which was then used to inoculate a microfluidic molecular device (PIE; GenePOC) with 150 μl of buffered sample. The PIEs were then added to the Revogene instrument, which can hold up to 8 PIEs and performs nucleic acid extraction followed by amplification and detection of the target sequence using rtPCR. The Revogene C. difficile test employs C. difficile primers and probe to detect a 263-bp target region of the toxin B (*tcdB*) gene of C. difficile. Results from the rtPCR were interpreted by the Revogene software and determined to be positive or negative for *tcdB*.

### SOC testing.

SOC test results were also collected for each specimen. The SOC test methods used in this study included the Xpert C. difficile assay (sites 2, 3, and 7), the BD MAX Cdiff assay (sites 1, 4, 6, and 7), the Illumigene C. difficile assay (site 4), the BD GeneOhm Cdiff assay (site 5), and the Luminex xTAG gastrointestinal pathogen panel (site 4). Site 1 performed BD MAX Cdiff testing followed by enzyme immunoassay testing (TechLab CDiff Quik Chek) if BD MAX Cdiff test results were positive. All testing was performed by following the laboratories’ policies, based on the manufacturers’ package inserts.

### Statistical analysis.

Results from the Revogene C. difficile assay were compared to those of direct and enriched cell culture. Performance characteristics, including positive percent agreement (PPA) and negative percent agreement (NPA), were calculated using standard methods. Ninety-five percent confidence intervals (CIs) were established by using a binomial expansion.

## RESULTS

### Specimen characteristics.

A total of 2,461 specimens were enrolled and tested with combined C. difficile culture methods, to characterize the performance of the Revogene C. difficile assay. Specimens were obtained from a range of age groups, including <2 years of age (*n* = 9), 3 to 18 years of age (*n* = 105), 19 to 60 years of age (*n* = 1,199), and >60 years of age (*n* = 1,148) (Table 1). The overall positivity rate for toxigenic C. difficile (*tcdB*) averaged 13.5%. The rates of toxigenic CDI varied among the age groups, from 11.6% to 31.1%, with the highest rate being observed for the <2-year-old population (*n* = 9). Carriage of C. difficile is common among infants, and samples generally should not be submitted for C. difficile testing (11).

**TABLE 1:** Toxigenic C. difficile prevalence in stool specimens, according to patient age

Patient age	No. of samples^*a*^					Sensitivity (%)	Specificity (%)	Positivity rate (%)
	TP	TN	FP	FN	Total			
<2 yr	3	6	0	0	9	100	100	33.3
3–18 yr	15	85	3	2	105	81.8	96.8	17.1
19–60 yr	120	1,022	26	31	1,199	83.7	97.3	12.6
>60 yr	145	956	30	17	1,148	90.9	97.3	14.1
Total	283	2,069	59	50	2,461	85.0	97.2	13.5

a Values are based on comparisons of Revogene and combined TC results.

### Comparison of Revogene C. difficile testing and the reference method for identification of toxigenic C. difficile.

The overall PPA and NPA for the Revogene C. difficile assay, compared to the combined reference method, were 85.0% and 97.2%, respectively (Table 2). All sites displayed Revogene *tcdB* NPA of >91% and PPA of >83% except for site 6, which had a lower PPA of 74.0%. Of the 2,461 specimens enrolled, 59 were positive by the molecular assay and negative by the reference method (false-positive [FP] samples), and 50 were negative by the molecular assay but positive by the reference method (false-negative [FN] samples).

**TABLE 2:** Performance of the Revogene C. difficile assay, compared to combined TC

Site and specimen type	No. of samples					PPA (95% CI) (%)	NPA (95% CI) (%)
	TP	TN	FP	FN	Total		
Site 1							
Fresh	14	63	6	2	85	87.5 (60–98)	91.3 (81–96)
Frozen	25	181	6	1	213	96.1 (78–100)	96.8 (93–99)
Total	39	244	12	3	298	92.8 (79–98)	95.3 (92–97)
Site 2							
Fresh	18	135	2	3	158	85.7 (63–96)	98.5 (94–100)
Frozen	41	225	4	6	276	87.2 (73–95)	98.2 (95–99)
Total	59	360	6	9	434	86.8 (76–93)	98.4 (96–99)
Site 3							
Fresh	6	72	1	0	79	100 (52–100)	98.6 (92–100)
Frozen	18	255	7	2	281	90.0 (67–98)	97.3 (94–99)
Total	24	327	8	2	361	92.3 (73–99)	97.6 (95–99)
Site 4							
Fresh	21	115	5	4	145	84.0 (63–95)	95.8 (90–98)
Frozen	18	102	3	1	124	94.7 (72–100)	97.1 (91–99)
Total	39	217	8	5	269	88.6 (75–96)	96.4 (93–98)
Site 5							
Fresh	0	0	0	0	0	NA^*a*^	NA
Frozen	13	171	5	0	189	100 (72–100)	97.1 (93–99)
Total	13	171	5	0	189	100 (72–100)	97.1 (93–99)
Site 6							
Fresh	16	148	5	7	176	69.6 (47–86)	96.7 (92–99)
Frozen	41	251	6	13	311	75.9 (62–86)	97.7 (95–99)
Total	57	399	11	20	487	74.0 (63–83)	97.3 (95–98)
Site 7							
Fresh	16	131	2	5	154	76.2 (52–91)	98.5 (94–100)
Frozen	36	220	8	5	269	87.8 (73–95)	96.5 (93–98)
Total	52	351	10	10	423	83.9 (72–91)	97.2 (95–98)
Total fresh	91	664	20	22	797	80.5 (72–87)	97.1 (95–98)
Total frozen	192	1,405	39	28	1,664	87.3 (82–91)	97.3 (96–98)
Study total	283	2,069	59	50	2,461	85.0 (80–88)	97.2 (96–98)

a NA, not applicable.

The specimens tested consisted of 32.4% fresh specimens (*n* = 797) and 67.6% retrospectively collected frozen (−80^0^C) specimens (*n* = 1,664) (Table 2). The single freeze-thaw cycle had no significant effect on the NPA (97.1% versus 97.3%) or the positivity rate (12.0% versus 12.0%). There was a slight difference in PPA between fresh and frozen specimens (80.5% and 87.3%, respectively).

### Performance of Revogene C. difficile testing according to patient location.

Data were stratified based on patient location, i.e., inpatient, outpatient, long-term care facility, or emergency department (Table 3). Three true-negative (TN) specimens were removed from this analysis because the location was unknown. The rates of *tcdB* identification varied somewhat among patients from the various locations, with specimens from emergency department patients having the lowest rate and specimens from long-term care facility patients having the highest rate (8.0% and 30.3%, respectively) (Table 3). Most specimens originated from hospitalized patients (inpatients) (*n* = 1,743 [70.8% of total specimens]), while specimens from patients in long-term care facilities were the least represented among the enrolled specimens (*n* = 61 [2.5% of total specimens]). There were ranges of PPA (82.7% to 88.9%) and NPA (92.5% to 97.5%) values. The only significant difference observed based on patient location was that testing of specimens from long-term care facility patients displayed lower NPA, which may be due to the small number of specimens tested (*n* = 61).

**TABLE 3:** Performance of the Revogene C. difficile assay according to patient location

Patient type	No. of samples					Prevalence (%)	PPA (95% CI) (%)	NPA (95% CI) (%)
	TP	TN	FP	FN	Total			
Inpatient	171	1,498	42	32	1,743	11.6	84.2 (78–89)	97.2 (96–98)
Outpatient	69	331	9	11	420	19.0	86.2 (76–93)	97.3 (95–99)
Long-term care facility	17	39	3	2	61	31.1	89.4 (65–98)	92.8 (79–98)^*a*^
Emergency department	26	198	5	5	234	13.2	83.8 (65–94)	97.5 (94–99)
Total	283	2,066^*b*^	59	50	2,458^*b*^	13.5	85.0 (80–88)	97.2 (96–98)

a Significantly different from other patient types, based on 95% CI.

b Three specimens with unknown patient locations were excluded from the analysis.

### Comparison to SOC testing and discrepancy analysis.

Of the 2,461 specimens enrolled in the study, 2,176 specimens (88.4%) had SOC test results to compare with the Revogene C. difficile assay results (Table 4). Data from SOC testing at site 4 were not available to compare with Revogene C. difficile assay results. Most of the SOC testing was performed with the BD MAX Cdiff assay (*n* = 941) or the Xpert C. difficile assay (*n* = 791). Positivity rates varied among the site-specific testing methods, being highest for BD MAX Cdiff testing (18.0%) and lowest for TechLab CDiff Quik Chek testing (6.3%). PPA and NPA, compared to SOC test results, ranged from 86.4% to 100% and from 94.5% to 99.2%, respectively.

**TABLE 4:** Performance of the Revogene C. difficile assay, compared to site SOC testing

Assay type	No. of samples					Prevalence (%)	PPA (95% CI)	NPA (95% CI)
	TP	TN	FP	FN	Total			
Revogene C. difficile	283	2,069	59	50	2,462	13.0	85.0 (80–88)	97.2 (96–98)
Xpert C. difficile	81	689	12	9	791	11.4	90.0 (81–95)	98.3 (97–99)
BD MAX Cdiff	147	765	6	23	941	18.0	86.4 (80–91)	99.2 (98–100)
TechLab CDiff Quik Chek	14	223	13	2	252	6.3	87.5 (60–98)	94.5 (90–97)
BD GeneOhm Cdiff	13	169	6	0	188	6.9	100 (72–100)	96.6 (92–98)
Total, excluding Revogene	255^*a*^	1,846^*a*^	37^*a*^	34^*a*^	2,172^*a*^	13.3	88.2 (84–92)	98 (97–99)

a A total of 289 specimens without site SOC results were excluded from the analysis.

Of the 109 TC-discrepant specimens, 96 had SOC test results (45 FN samples and 51 FP samples). Compared to SOC testing, 64.4% of the FN specimens (29/45 specimens) were negative by SOC testing. Additionally, results for 51.0% of the FP specimens (26/51 specimens) agreed with the Revogene C. difficile assay results. Collectively, use of the Revogene C. difficile molecular assay, in comparison to all of the SOC testing at the enrollment sites, resulted in increased PPA (88.2%) (Table 4).

## DISCUSSION

The literature demonstrates variability in the performance characteristics of currently available molecular assays for C. difficile detection, with sensitivities and PPA, compared to TC, ranging from 81.6% to 100% (9, 12–17). This wide range is likely due to a variety of factors, including bacterial load, targeted patient populations, and the sensitivity of the comparative culture method, since there is no agreed-upon or approved standard method. Utilization of molecular assays has suggested that many “gold standard” methods miss samples due to low *tcdB* copy numbers that are below the limit of detection for the culture and antigen methods (14, 15, 18). The PPA observed in our study (85%) was comparable to those in other studies addressing the performance of molecular detection of C. difficile, including studies with the Xpert C. difficile assay (82.8% or 90%) (14, 15), the BD MAX Cdiff assay (81.6% to 89.3%) (12, 14, 15, 17), the Simplexa C. difficile assay (87.4%) (17), and the Illumigene C. difficile assay (82.4% or 86.7%) (15, 18). However, other studies regularly observed sensitivities and PPA over 90% for C. difficile molecular assays, including the Xpert C. difficile assay (93.5% to 100%) (9, 12, 16, 17), the Illumigene C. difficile assay (91.8%) (19), the BD GeneOhm Cdiff assay (95.5%) (18), and the Verigene CDF assay (5.2%) (17). Importantly, it must be mentioned that none of the aforementioned studies determined the performance of the molecular assays in comparison to a reference method that combined direct culture and enriched culture, as employed by this study. The reference method used to evaluate a new assay can have large effects on overall performance, which is likely the cause of variability between studies.

A weakness of this study was the lack of semiquantitative data for C. difficile culture, to enable a comparison with FN samples and specimens with low bacterial loads. The lower PPA found in this study is likely due to the rigorous reference testing used for the evaluation. The reference method included direct TC testing and enrichment broth culture, which promoted cellular growth and thus increased low levels of toxin. Among FN specimens, 41/50 specimens (82%) were positive only when enrichment broth culture was performed; compared to direct TC only, the PPA increased from 85.0% to 95.3% (data not shown). The latter PPA correlates better with those of other molecular assays, because the same methodology of direct culture was used for the comparison (12, 15, 16, 18, 19). There are at least two explanations for the difference between enriched culture and direct culture. Because enrichment broth culture was used to detect low levels of organisms from specimens, some FN results might be due to organisms being present at concentrations below the limit of detection for both the Revogene C. difficile assay and direct plating. Alternatively, because enrichment broth culture requires only a few CFU or spores to grow to high bacterial counts, contamination could have occurred between inoculation of specimens, resulting in a FN molecular test report. Low levels of starting organisms likely played a role, as enrichment broth culture detected an additional 59 true-positive (TP) specimens that direct culture alone would have missed.

A strength of this study was the multicenter approach, with specimens collected throughout North America. While no ribotyping was performed, the C. difficile strains found in this study are most likely a diverse representation of C. difficile strains, providing a more realistic comparison to the highly sensitive TC. The potentially diverse strains represented in this study might have been a factor in the level of sensitivity observed (8, 16). A multicenter study utilizing the Xpert C. difficile molecular assay found that the C. difficile subtype played a role in the detection of *tcdB*, as sensitivities ranged from 75% to 100% among 8 ribotypes (16).

The Revogene molecular assay for *tcdB* detection in C. difficile requires <5 min of hands-on time and produces results with a turnaround time of 70 min, with a high negative predictive value. Additionally, the Revogene assay can accommodate up to 8 microfluidic cartridges (PIEs), to facilitate batch testing of 1 to 8 specimens per run. This instrumentation would be suitable for any laboratory that requires rapid results from rtPCR technology and the flexibility for small-batch testing. The incorporation of a molecular assay into a multistep algorithm in testing for C. difficile can be justified by earlier reporting of CDI cases, thus preventing further spread of nosocomial CDI, with shortened length of stay for CDI patients (8, 16). Additionally, the high negative predictive value and reduced turnaround time with molecular testing can reduce the practice of multiple test ordering, as well as the total numbers of C. difficile toxin assays ordered (12). The impact of the Revogene C. difficile assay on patient management will likely be similar to those of other molecular assays; when combined with proper clinical assessment of patients at risk of CDI or used as a frontline test in a multistep testing approach, it could improve patient management, infection control, and the reliability of surveillance data (10, 12, 13). The IDSA guidelines enforce this view, as they specifically recommend a stool toxin test, as well as a molecular assay or GDH assay, as part of a multistep testing algorithm if no preestablished institutional criteria for patient stool submission are in place. Because this is the first study to directly evaluate the performance of the Revogene C. difficile assay, compared with TC, additional studies should be performed to fully assess the clinical performance of this assay, relative to other commercially available molecular tests.
